# Therapy-Related-Myeloid-Neoplasm-Risk Score: a convenient score for therapy-related myeloid neoplasms risk assessment in adult cancer patients

**DOI:** 10.1093/jncics/pkaf087

**Published:** 2025-09-16

**Authors:** Abhay Singh, Megan M Herr, Rahul Mishra, Rusina Karia, Theresa Hahn, Swapna Thota

**Affiliations:** Department of Hematology Oncology, Taussig Cancer Institute, Cleveland Clinic, Cleveland, OH, United States; Department of Medicine, Roswell Park Comprehensive Cancer Center, Buffalo, NY, United States; Department of Internal Medicine, Anne Arundel Medical Center, Annapolis, MD, United States; Department of Internal Medicine, University Hospitals Community Consortium, Geauga, OH, United States; Department of Cancer Prevention and Control, Roswell Park Comprehensive Cancer Center, Buffalo, NY, United States; Division of Hematology/Oncology, University of Tennessee Health Science Center, Memphis, TN, United States

## Abstract

**Background:**

A prediction model for estimating risk of therapy-related myeloid neoplasms (tMNs), a late effect with a high mortality after chemotherapy and/or radiation, is currently unavailable. Ability to predict risk at initial cancer presentation can be key for early detection and risk mitigation.

**Methods:**

Using SEER-Medicare linked database, 970 390 adults diagnosed with first primary cancer from 2000 to 2011 (with follow-up through 2015) were selected. The sample was divided into training (*n* = 582 234) and validation cohorts (*n* = 388 156). Various tMN risk factors were used for the development of tMN prediction model: the Therapy-Related Myeloid Neoplasm Risk Score (TMNRS). TMNRS was created as a simple arithmetic sum of independent predictors of tMN weighted according to the adjusted hazard ratio from the Cox proportional hazards analysis.

**Results:**

In addition to the known risk factors of chemotherapy and radiation exposure, history of autoimmune disease and granulocyte-colony stimulating factor exposure emerged as consistent predictors of tMN after each of the 5 cancers in the study. Cancer survivors were categorized into distinct risk groups with variable risk of tMN.

**Conclusion:**

TMNRS provides a simple and convenient office-based mechanism to identify solid cancer patients at variable risks of tMN development. This risk assessment tool provides preliminary insights that may contribute to future research on the management of patients, particularly those receiving adjuvant therapies. Further investigation is required to fully evaluate its clinical utility and potential effects on patient care.

## Introduction

In the United States, there are an estimated 18.1 million cancer survivors. This number is projected to increase to 22.5 million by 2032.^1^ Long-term cancer survivors encounter late health effects from their cancer diagnosis and its treatment, requiring continued evaluation and care.^1^ Risk prediction models of late effects of cancer treatments such as cardiac dysfunction or ovarian failure assist physicians and aid in optimizing long-term care.^2^ A prediction model to estimate risk of therapy-related myeloid neoplasms (tMNs) is not available. tMN is a late effect after chemotherapy and/or radiation exposure, which remain key cancer treatments for several primary solid tumors,^3^ with a high mortality.^4^ The ability to predict tMN risk at initial cancer presentation can be key for early detection and risk mitigation.

To be clinically pragmatic, a risk prediction tool must be simple and easily applicable. A tool that predicts tMN risk should make use of clinical data that are routinely available at initial presentation in the treating oncologist’s office. However, to perform accurately, the tool must use data that take into account the complex profile of cancer patients with multiple risk factors. tMNs are particularly detrimental in patients older than 65 years of age because of several factors. The incidence of acute myeloid leukemia (AML) and myelodysplastic syndrome (MDS) increases significantly with age, reflecting a higher baseline vulnerability of older patients to hematologic malignancies. Second, outcomes for AML and MDS are generally worse in this age group compared with younger patients, largely due to age-related comorbidities, reduced tolerance to aggressive treatments, and the presence of high-risk genomic aberrations. Consequently, a prediction tool focusing on an older patient population with tMN is critical as they represent a group with inherently poorer prognoses and greater therapeutic challenges. Toward that end, in the current investigation we used the merged Surveillance, Epidemiology, and End Results (SEER)–Medicare database as the targeted cohort, to elucidate the association of multiple risk factors in predicting tMN risk in elderly long-term cancer survivors.

Several risk factors exist for AML and MDS, including age, smoking, genetic inheritance, cytotoxic or immunosuppressive treatments.^5^ Clonal hematopoiesis of indeterminate potential (CHIP) is a novel risk factor for overt myeloid neoplasms, is enriched among older people and cancer survivors, and is associated with atherosclerotic cardiovascular disease^6-12^ and other nonhematological disorders.^13^ MDS has been associated with history of autoimmune disease although it is not clear if the disease or its treatment leads to t-MDS.^14^^,^^15^ Granulocyte-colony stimulating factor (G-CSF) exposure has been implicated in tMN development.^16-18^ We considered all of these factors along with several well-established factors for the development of our tMN prediction model, called Therapy-Related Myeloid Neoplasm Risk Score (TMNRS).

## Methods

### Population

We conducted a population-based retrospective cohort study using the SEER–Medicare linked database; 970 390 adults diagnosed with first primary cancer from 2000 to 2011 (with follow-up through death or 2015, whichever occurred earlier) were selected from the Patient Entitlement and Diagnosis Summary File (PEDSF; see Table S1 for diagnoses definitions). The sample was divided into training (*n* = 582 234) and validation cohorts (*n* = 388 156). Patients were followed through 2015 to investigate tMN risk using the PEDSF file. A 1-year latency period between their primary cancer and tMN was required to avoid surveillance bias. In this Medicare sample with eligibility starting at age 65, we required 1-year minimum follow-up for all patients; therefore, the age at first cancer was limited to 66 years. The maximum age was 85 years to account for decreased surveillance of individuals older than 85 years. Individuals who were diagnosed with their first cancer at autopsy or by death certificate were excluded.

The presence of medical conditions identified a priori were assessed using Medicare claims. Individuals were classified as having a medical condition of interest if they had at least 1 hospital claim, or 2 physician/outpatient claims at least 30 days apart at least 2 months prior to tMN diagnosis. We excluded medical conditions/exposures that occurred within 2 months of tMN diagnosis to avoid biases from differential assessment of exposure status. Patients were excluded if tMN or initial cancer diagnosis month was unknown. Medical conditions and covariates in model construction included age, gender, race, socioecomonic status, prior receipt of chemotherapy and radiotherapy, history of transplant, type of transplant, prior history of cardiovascular disease (stroke, heart disease, and other vascular diseases), history of autoimmune disease, diagnosis of hypertension, diabetes, history of infections, and exposure to growth factors (see Tables S2 and S3 for diagnoses definitions). Autoimmune disease was categorized as acute (acute presentation requiring short course of immunosuppressive therapy) or chronic (requiring continuous maintenance immunosuppressive therapy for a prolonged period over multiple years) (Table S4). Race/ethnicity were hierarchically defined as Hispanic (any race), non-Hispanic White (White), non-Hispanic Black (Black), and non-Hispanic Other (Other). Socioeconomic status was assessed from the PEDSF file by using median family or household income and percentage of persons below poverty variables based on patient’s census tract.

### Statistical analyses

Descriptive analyses were performed to compare risk factors for tMN and factors associated with clonal hematopoiesis including infections, autoimmune disease, presence of cardiovascular disease–associated factors, and tMN by χ^2^ tests. Significant factors associated with tMN and clonal hematopoiesis in the univariable analyses were included in the multivariable model using backward elimination. Multivariable analyses were also adjusted for latency, duration of part A, part B non-HMO coverage, and number of physician visits, hospital, or outpatient claims, if associated with tMN. Of the factors considered significant in the univariable model, acute and chronic autoimmune disease and infections were entered into the multivariable model as time-dependent covariates. Cardiovascular disease was not included as a time-dependent covariate because almost 75% of patients had cardiovascular disease at the time of the first cancer diagnosis. The proportional subdistribution hazard model of Fine and Grey^19^ was finalized using a backward elimination technique with death from any cause as a competing risk, and statistical significance was set at *P* < .01 to control for multiple comparisons. Model fit was assessed using the Akaike Information Criterion (AIC), and the model with the smallest AIC was selected as the final model even if the parameters were no longer statistically significant at *P* = .01.

Stratified cancer-specific tMN risk prediction models were built for cancers with a sufficient number of tMN cases (>150) resulting in models for 5 primary cancers (prostate, gastrointestinal [GI], breast, lung, and bladder). The aforementioned statistical methods were replicated for each individual prediction model in the training cohort. Each cancer-specific final model was then tested in the validation cohort. The factors that had the same direction and magnitude of effect with a significant *P* value were retained in the final tMN risk score models.

The TNMRS was created separately for each first primary cancer as a simple arithmetic sum of independent predictors of tMN weighted according to the hazard ratio (HR) from the multivariable Cox proportional hazards model based on each cancer-specific final model in the training cohort. Point values were assigned to each risk predictor based on this model (Table 1): 0.5 points for HR = 1.0-1.9 (1.0 being the reference group), 1 point for HR = 2.0-2.9, 2 points for HR = 3.0-3.9, and so on. Patients were categorized into cancer-specific risk groups (low, intermediate, and high) based on their cancer-specific risk score in the training cohort (details in cancer-specific results sections), which were tested in the validation cohort. Further details can be found in the Supplemental Methods.

**Table 1.** Risk of tMN after first primary solid cancer diagnosed 2000-2011 using SEER–Medicare.

	Female breast cancer		Prostate cancer		Gastrointestinal cancer		Lung cancer		Bladder cancer	
	HR (95% CI)	*P*	HR (95% CI)	*P*	HR (95% CI)	*P*	HR (95% CI)	*P*	HR (95% CI)	*P*
**Chemotherapy or radiation and growth factor**		<.0001		<.0001		<.0001		<.0001		<.0001
No chemo/rad (± growth factor)	Referent		Referent		Referent		Referent		Referent	
Chemo/rad but no growth factor	1.91 (1.49 to 2.44)		2.91 (2.50 to 3.38)		1.06 (0.84 to 1.36)		0.92 (0.65 to 1.31)		2.01 (1.50 to 2.69)	
Chemo/rad/growth factor	6.68 (4.81 to 9.27)		3.94 (2.26 to 6.87)		3.24 (2.19 to 4.80)		4.53 (3.11 to 6.59)		1.70 (0.70 to 4.11)	
**Acute autoimmune disorder**		<.0001		<.0001		.0003		.10		<.0001
No autoimmune disorder	Referent		Referent		Referent		ref		Referent	
Autoimmune disorder	1.78 (1.34 to 2.36)		2.32 (1.92 to 2.80)		1.73 (1.28 to 2.34)		1.44 (0.93 to 2.23)		2.33 (1.56 to 3.48)	
**Chronic autoimmune disorder**		<.0001		<.0001		<.0001		<.0001		.003
No autoimmune disorder	Referent		Referent		Referent		Referent		Referent	
Autoimmune disorder	1.94 (1.55 to 2.43)		1.59 (1.36 to 1.87)		1.72 (1.35 to 2.19)		2.42 (1.75 to 3.34)		1.69 (1.20 to 2.38)	
**Year of first primary cancer diagnosis**		<.0001		<.0001		0.008				.0014
2008-2011	Referent		Referent		Referent				Referent	
2004-2007	2.23 (1.72 to 2.90)		1.55 (1.31 to 1.84)		1.40 (1.05 to 1.85)				2.06 (1.39 to 3.05)	
2000-2003	1.29 (0.97 to 1.72)		1.30 (1.08 to 1.56)		1.56 (1.17 to 2.07)				1.66 (1.10 to 2.52)	
**Stage of first primary cancer diagnosis**				<.0001		<.0001		.0003		
Advanced stage			Referent		Referent		Referent			
Localized/regional/unknown			3.47 (2.13 to 5.63)		3.44 (2.08 to 5.69)		2.04 (1.38 to 3.01)			
**Census Tract Poverty Indicator**				<.0001						.0008
5%-100%			Referent						Referent	
0 to <5%, unknown			1.30 (1.14 to 1.49)						1.64 (1.23 to 2.18)	
**Age at first primary cancer**				<.0001						
<70 years			Referent							
70 to <75 years			1.80 (1.50 to 2.15)							
≥75 years			3.33 (2.79 to 3.97)							
**Sex**						<.0001		.0058		.06
Female		-	-		Referent		Referent		Referent	
Male					1.95 (1.55 to 2.44)		1.52 (1.13 to 2.04)		1.44 (0.99 to 2.08)	
**Race**				.001		.03		.2		
Black and Unknown			Referent		Referent		Referent			
Other			1.46 (1.17 to 1.83)		1.68 (1.06 to 2.67)		1.60 (0.82 to 3.12)			
**Infection**		<.0001		.01						
No infection	Referent		Referent							
Infection	1.72 (1.33 to 2.22)		1.23 (1.05 to 1.45)							
**Cardiovascular disease**				.002						<.0001
Cardiovascular disease			Referent						Referent	
No cardiovascular disease			1.30 (1.10 to 1.53)						2.12 (1.49 to 3.00)	

Abbreviations: CI = confidence interval; HR = hazard ratio; SEER = Surveillance, Epidemiology, and End Results; tMN = therapy-related myeloid neoplasm.

All variables were considered for all models (with the exception of the sex type for female breast cancer and prostate cancer). Model fit was assessed by improvement in Akaike Information Criterion. These models were additionally controlled for the average annual number of physician visits and duration of Medicare coverage, if statistically significant.

Cumulative incidence curves of time to tMN diagnosis by first primary cancer were calculated and stratified by year of first primary cancer diagnosis. Data were analyzed using SAS 9.4 (Cary, NC).

## Results

### Study population

Sample characteristics in the training (*N* = 582 234) and validation (*N* = 388 156) cohorts are shown in Table S5 and are evenly distributed. In both cohorts, women comprised 43% of the sample, 85% was White, and 9% were Black. The most common first primary cancer in both cohorts was prostate (31%), followed by GI (19%), breast (18%), lung (10%) and bladder (7%). Six percent of the cohort had a history of an acute autoimmune disease, 13% had a history of a chronic autoimmune disease, and 3% had exposure to G-CSF.

### Predictors of tMN

**Figure 1.**
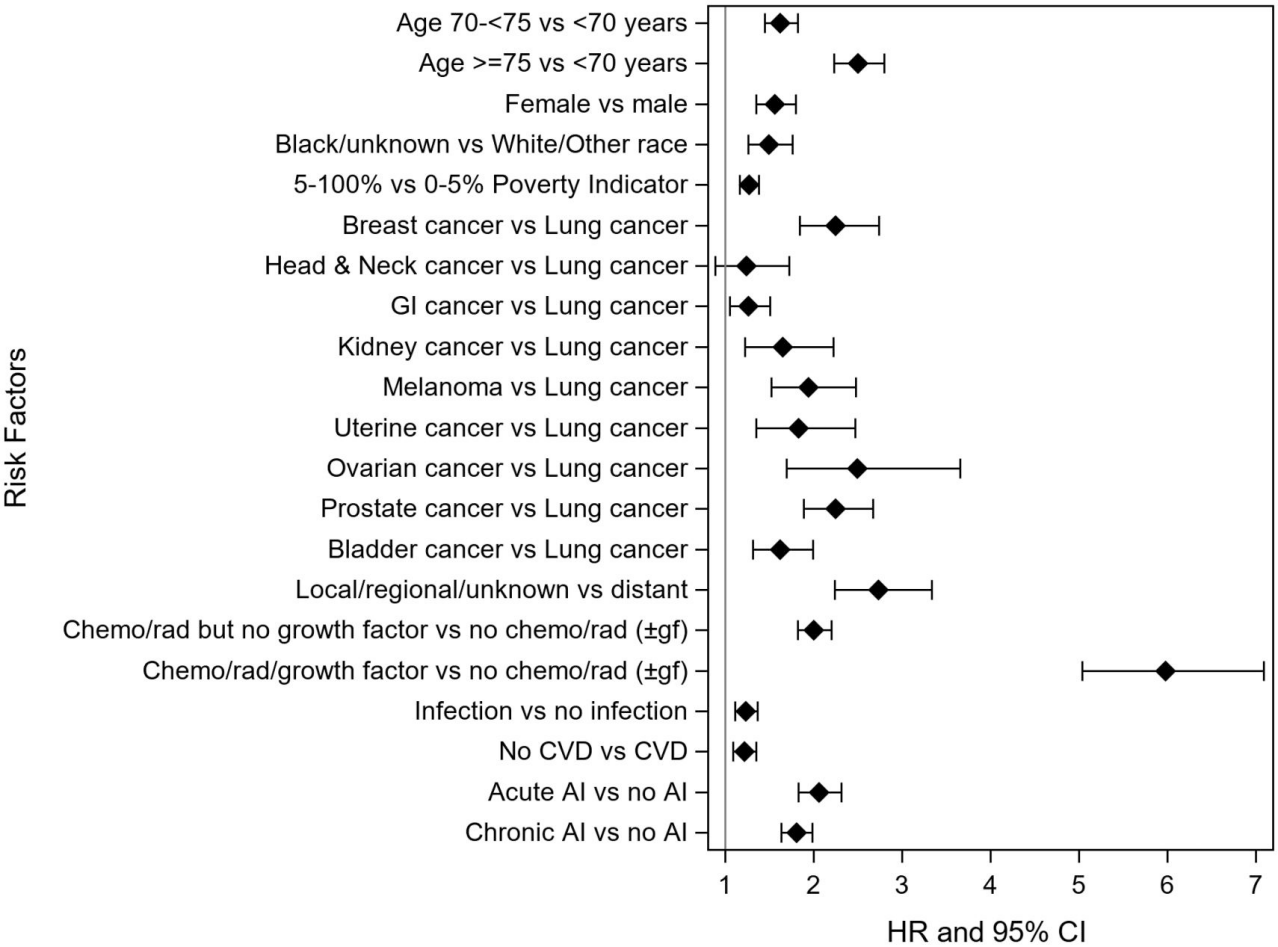
Forest plot demonstrating risk of therapy-related myeloid neoplasm after first primary solid cancer diagnosed 2000-2011 and other clinical variables, using SEER-Medicare.

A total of 2426 tMN cases were identified in the training cohort (Table S6). After initial chemotherapy, radiation, and growth factor for the first primary solid cancer, the occurrence of tMN was statistically significantly higher compared to those who did not receive chemotherapy or radiation (± growth factor) in their initial regimen (HR = 5.98, 95%CI = 5.04 to 7.09, *P* < .0001; Figure 1). History of autoimmune diseases also indicated a significantly higher likelihood of subsequent tMN compared with no history of autoimmune disease (acute: HR = 2.05, *P* < .0001; chronic: HR = 1.80, *P* < .0001). Risk of tMN was higher in all other cancers compared with first primary lung cancer (*P* < .0001), although the confidence bound for risk of tMN after Head and Neck cancer crossed 1.0. Risk of tMN was also higher for men (*P* < .0001), patients of other race/ethnicity (*P* < .0001), patients from a high-poverty census tract (*P* < .0001), earlier stage (*P* < .0001), and no cardiovascular disease (*P* = .0004), infection at least 2 months prior to tMN (*P* = .0001; Figure 1; Table S6).

Multivariable analyses were then stratified by first primary cancer focusing on those cancers with at least 150 tMN cases.

#### Female breast cancer

After first primary female breast cancer, chemotherapy ± radiation (without G-CSF) significantly increased risk of tMN development compared to no chemotherapy or radiation (±G-CSF; HR = 1.91, 95% CI = 1.49 to 2.4, *P* < .0001; Table 1). This risk was much higher (HR = 6.68, 95% CI = 4.81 to 9.27, *P* < .0001) after chemotherapy ± radiation with G-CSF. Other predictors identified in the univariate analysis included history of an acute or chronic autoimmune disease, and infections. The complete model validated.

#### Prostate cancer

The risk of tMN after first primary prostate cancer was also significantly elevated after chemotherapy ± radiation with (HR = 3.94, 95% CI = 2.26 to 6.87, *P* < .0001) and without (HR = 2.91, 95% CI = 2.50 to 3.38, *P* < .0001; Table 1) G-CSF exposure. Other tMN predictors included age at initial diagnosis, stage at diagnosis, race/ethnicity, history of acute or chronic autoimmune disease, and prior history of infections. Prior history of cardiovascular disease (CVD) (No CVD: HR = 1.30, 95% CI = 1.10 to 1.53, *P* = .002) and advanced-stage (early-stage disease: HR = 3.47, 95% CI = 2.13 to 5.63) were associated with decreased risk of subsequent tMN. These findings are likely functions of survival bias, that is, patients with CVD have shorter survival compared with those without CVD; therefore, CVD patients may not survive long enough to develop tMN. In the validation model, poverty and race were not statistically significant.

#### GI cancer

The risk of tMN after initial GI cancer was significantly elevated after chemotherapy ± radiation exposure with G-CSF (HR = 3.24, 95% CI = 2.19 to 4.80, *P* < .0001); however, there was no increased risk in the absence of G-CSF exposure (HR = 1.06, 95% CI = 0.84 to 1.36; Table 1) compared to no chemotherapy and radiation (±G-CSF). Other predictors include early stage at initial diagnosis, sex, race/ethnicity, and history of an acute or chronic autoimmune disease. The model validated.

#### Lung cancer

The risk of tMN after first primary lung cancer was significantly elevated after chemotherapy ± radiation exposure with G-CSF (HR = 4.53, 95% CI = 3.11 to 6.59, *P* < .0001); however, there was no association in the absence of G-CSF exposure (HR = 0.92, 95% CI = 0.65 to 1.31; Table 1). History of a chronic autoimmune disease (HR = 2.42, 95% CI = 1.75 to 3.34, *P* < .0001) was a significant predictor of tMN after lung cancer as well as male sex (HR = 1.52, 95% CI = 1.13 to 2.04, *P* = .0058), although both of these risk factors did not validate.

#### Bladder cancer

The risk of tMN was significantly higher after chemotherapy ± radiation exposure without G-CSF (HR = 2.01, 95% CI = 1.50 to 2.69, *P* < .0001; Table 1) after first primary bladder cancer. Other predictors of tMN included history of an acute or chronic autoimmune disease, lack of cardiovascular disease, poverty, and male sex, although poverty did not validate.

Overall, we noted that in addition to the known risk factors of chemotherapy ± radiation and G-CSF exposure, a history of autoimmune disease emerged as consistent predictors for all 5 primary cancers. History of autoimmune disease maintained statistically significant even in the absence of chemo-radiation therapy exposure.

### Therapy-Related Myeloid Neoplasm Risk Score

The TMNRS, developed from the individual cancer-specific hazard ratios from the multivariable Cox proportional hazards model, is a simple arithmetic sum of significant independent predictors of subsequent tMNs. Cancer survivors were categorized into distinct risk groups with the proportion of patients with tMN ranging from 0.24% to 1.11% after first prostate cancer, 0.17% to 1.47% after first GI cancer, 0.26% to 1.66% after first female breast cancer, 0.12% to 1.21% after first lung cancer, and 0.24% to 1.34% after first bladder cancer in the training cohort (Table 2). These results in the validation cohort were similar (Table 2) with the proportion of patients with tMN ranging from 0.24% to 1.16% after first prostate cancer, 0.23% to 1.43% after first GI cancer, 0.27% to 1.49% after first female breast cancer, 0.13% to 1.03% after first lung cancer, and 0.25% to 1.47% after first bladder cancer.

**Table 2.** Incidence of therapy-related myeloid neoplasm, overall and stratified by risk groups in the training and validation cohorts by first primary cancer.

	Breast	Prostate	Gastrointestinal	Lung	Bladder
	*n* = 173 754	*n* = 298 934	*n* = 184 811	*n* = 100 636	*n* = 72 333
No. of tMN cases	694	1685	601	300	322
Overall proportion of tMN	0.40%	0.60%	0.30%	0.30%	0.40%
Training					
Low	0.26%	0.24%	0.17%	0.12%	0.24%
Intermediate	0.46%	0.53%	0.44%	0.30%	0.52%
High	1.66%	1.11%	1.47%	1.21%	1.34%
					
Validation					
Low	0.27%	0.24%	0.23%	0.13%	0.25%
Intermediate	0.49%	0.55%	0.45%	0.29%	0.53%
High	1.49%	1.16%	1.43%	1.03%	1.47%

Abbreviation: tMN = therapy-related myeloid neoplasm.

## Discussion

Using the significant factors from a multivariable analysis in a large and diverse claims database of cancer survivors, we developed a convenient office-based clinical score that may be applied at the time of patient presentation to assess the risk of tMN development. The predictive capacity of this risk score was reliable in these specific cancer cohorts. Furthermore, the TMNRS performed well in a large validation dataset of cancer survivors.

As the prevalence of cancers survivors is on the rise in the United States,^20^ effective risk stratification at initial presentation for subsequent tMN development is integral to the management of cancer patients with shared decision making. This becomes particularly important among patients with early-stage cancers where adjuvant therapeutic modalities (chemotherapy and radiation) may only provide marginal survival benefits. Knowledge regarding subsequent risk of tMN development can have an immense impact on early therapeutic decision making. The risk of MN in older adults in the general population ranges from 0.007% to 0.036%,^21^ whereas risk of tMN after a first primary ranges from 0.12% to 0.26% in low-risk patients to 1.11% to 1.66% in high-risk patients. Therefore, tools that enhance the treating oncologist’s ability to rapidly and accurately assess tMN risk are of significant interest. Toward this end, recent attempts have been made to develop risk stratification schemes, which may be calculated at the bedside using clinical parameters.^22^ The model was developed using hematological parameters in patients presenting to hematology clinics for cytopenias, derived from only 200 patients. The study reported a high crude incidence of tMN at 51%, highlighted as a limitation by authors of the study. Models that have studied large population databases and integrated weighting information from multivariate analysis in a manner similar to our TMNRS are lacking.

The clinical data included in the TMNRS are all routinely collected at initial presentation. The majority of the variables included in our model were independent predictors of long-term tMN development. Some factors associated with CHIP (a pre-leukemic state)^8^ were tested in the model.^7^^,^^23-25^ Notably, the association between autoimmune disease and increased tMN risk was consistently elevated across all first primary cancers. We intentionally differentiated between acute and chronic autoimmune diseases (details in Table S4) as they can differ based on duration, severity, treatment, and clinical course. Acute autoimmune disorders exhibit sudden and severe immune responses that may resolve quickly after short courses of immunosuppression. Our results indicated that increased risk of tMN occurred after prior diagnosis of both acute and chronic autoimmune diseases with 1 exception (lung cancer was associated only with chronic autoimmune disease). These findings speak to the larger role of underlying immune dysregulation in tMN pathogenesis than the use of prolonged immunosuppressive therapies to treat chronic autoimmune diseases. Of note, elevated tMN risk in those with prior history of autoimmune disease was evident even in the absence of chemotherapy or radiation therapy exposure.

Another novel predictor, G-CSF, emerged when used in combination with chemotherapy ± radiation therapy which significantly elevated the risk of tMN. At a molecular level, our group has shown that abrupt leukocytosis can lead to CHIP mutation propagation.^26^ Akin to that, we hypothesize that increased risk of tMN after G-CSF exposure is due to CHIP mutation expansions that persist and consequently lead to tMN. As such, our group is studying this at a molecular level in a cohort of cancer survivors.^27^ Overall, these readily available risk factors constitute a robust risk score that can be calculated at the bedside or in the office by a care provider with the aid of an online calculator. Risk prediction models for transformation from CHIP to overt myeloid neoplasm are now available.^28^ Combining TMNRS with molecular data in larger cohorts might further improve the model’s predictive accuracy. TMNRS provides a possibility to focus on risk factor mitigation strategies. Inclusion of molecular data and genetic characterization will further enable earlier and tailored prevention.^29^ TMNRS may also be used to design screening or prevention focused trials. A population at higher risk of harboring CHIP mutations can potentially be identified which provides opportunities for mutation screening via blood-based next-generation sequencing strategies to better inform prevention clinical trials. The high health-care-related economic burden in the United States has intensified the need for appropriate triage and clinical resource utilization. This strategy permits a financially viable testing strategy to screen cancer survivors at higher risk for harboring CHIP, which is currently employed at our center.^27^

The TMNRS has limitations. As our analysis was based on data sourced from the Medicare claims database, it is probable that our findings cannot be applied to a younger demographic. Second, our study involves incorporation of risk factors associated with CHIP; however, it lacks incorporation of CHIP mutations in the analysis. As CHIP mutation data are not currently available as standard of care for patients with an initial solid cancer diagnosis, our study provides an impetus to obtain such data in a research setting to further aid clinical decision making. Another novel predictor identified in our study that will need molecular characterization is exposure to G-CSF. Whether it is the dose-dense chemotherapy warranting the use of G-CSF (as neutropenic prophylaxis) that drives the tMN risk or is it the exposure to G-CSF that accentuates risk of transformation remains to be elucidated. Smoking and obesity are not accurately captured in the Medicare dataset, so we were unable to account for these known risk factors in our model. Last, our model demonstrated history of CVD and advanced stage disease as being protective for tMN development, which likely represents survival bias.

Overall, the TMNRS provides a simple and convenient mechanism to identify baseline differences in risk of tMN at initial presentation in solid cancer patients and offers an effective framework for analyses stratified by tMN development risk group. The TMNRS (available online; https://redcap.link/TMNRScalculator)^30^^,^^31^ uses readily available data and does not require invasive (bone marrow testing) or expensive (next-generation sequencing) strategies for tMN risk prediction. The score captures novel and important clinical information offered by a robust Cox proportional hazards model. This risk assessment tool is likely to be clinically useful in the clinical management of patients, especially those receiving adjuvant therapies, and will likely serve as a valuable aid in clinical research.

## Supplementary Material

pkaf087_Supplementary_Data

## Data Availability

The data used in this study were obtained from the SEER–Medicare linked database after application and approval from National Cancer Institute (NCI) SEER-Medicare. Access to these data is subject to restrictions and is available to researchers through application to the NCI. Due to the nature of the data agreements, the raw data cannot be made publicly available without prior NCI-SEER-Medicare permission.

## References

[1] Division of Cancer Control & Population Sciences, National Cancer Institute. Office of Cancer Survivorship. Statistics and graphs. Accessed March 8, 2025. https://cancercontrol.cancer.gov/ocs/statistics#stats

[2] Clark RA , Mostoufi-Moab S, Yasui Y, et al Predicting acute ovarian failure in female survivors of childhood cancer: a cohort study in the Childhood Cancer Survivor Study (CCSS) and the St Jude Lifetime Cohort (SJLIFE). Lancet Oncol. 2020;21:436-445. 32066539 10.1016/S1470-2045(19)30818-6 PMC7060129

[3] Morton LM , Dores GM, Schonfeld SJ, et al Association of chemotherapy for solid tumors with development of therapy-related myelodysplastic syndrome or acute myeloid leukemia in the modern era. JAMA Oncol. 2019;5:318-325. 30570657 10.1001/jamaoncol.2018.5625 PMC6439835

[4] McNerney ME , Godley LA, Le Beau MM. Therapy-related myeloid neoplasms: when genetics and environment collide. Nat Rev Cancer. 2017;17:513-527. 28835720 10.1038/nrc.2017.60 PMC5946699

[5] Vakiti A , Reynolds SB, Mewawalla P. Acute myeloid leukemia. In: StatPearls [Internet]. Treasure Island (FL): StatPearls Publishing; 2018. PMID: 29939652. 29939652

[6] Bewersdorf JP , Ardasheva A, Podoltsev NA, et al From clonal hematopoiesis to myeloid leukemia and what happens in between: will improved understanding lead to new therapeutic and preventive opportunities? Blood Rev. 2019;37:100587. 31400824 10.1016/j.blre.2019.100587

[7] Singh A , Balasubramanian S. The crossroads of cancer therapies and clonal hematopoiesis. Semin Hematol. 2024;61:16-21. 38403501 10.1053/j.seminhematol.2024.01.006

[8] Singh I , Singh A. Clonal hematopoiesis of indeterminate potential: current understanding and future directions. Curr Oncol Rep. 2023;25:539-547. 36928826 10.1007/s11912-023-01382-9

[9] Zink F , Stacey SN, Norddahl GL, et al Clonal hematopoiesis, with and without candidate driver mutations, is common in the elderly. Blood. 2017;130:742-752. 28483762 10.1182/blood-2017-02-769869 PMC5553576

[10] Jan M , Ebert BL, Jaiswal S. Clonal hematopoiesis. Semin Hematol. 2017;54:43-50. 28088988 10.1053/j.seminhematol.2016.10.002 PMC8045769

[11] Bolton KL , Ptashkin RN, Gao T, et al Cancer therapy shapes the fitness landscape of clonal hematopoiesis. Nat Genet. 2020;52:1219-1226. 33106634 10.1038/s41588-020-00710-0 PMC7891089

[12] Coombs CC , Zehir A, Devlin SM, et al Therapy-related clonal hematopoiesis in patients with non-hematologic cancers is common and associated with adverse clinical outcomes. Cell Stem Cell. 2017;21:374-382.e4. 28803919 10.1016/j.stem.2017.07.010 PMC5591073

[13] Jaiswal S. Clonal hematopoiesis and nonhematologic disorders. Blood. 2020;136:1606-1614. 32736379 10.1182/blood.2019000989 PMC8209629

[14] Al Ustwani O , Ford LA, Sait SJN, et al Myelodysplastic syndromes and autoimmune diseases—case series and review of literature. Leuk Res. 2013;37:894-899. 23692654 10.1016/j.leukres.2013.04.007 PMC3699612

[15] Hochman MJ , DeZern AE. Myelodysplastic syndrome and autoimmune disorders: two sides of the same coin? Lancet Haematol. 2022;9:e523-e534. 35772431 10.1016/S2352-3026(22)00138-7

[16] Lyman GH , Dale DC, Wolff DA, et al Acute myeloid leukemia or myelodysplastic syndrome in randomized controlled clinical trials of cancer chemotherapy with granulocyte colony-stimulating factor: a systematic review. J Clin Oncol. 2010;28:2914-2924. 20385991 10.1200/JCO.2009.25.8723

[17] Lyman GH , Dale DC. Long-term outcomes of myeloid growth factor treatment. J Natl Compr Canc Netw. 2011;9:945-952. 21900223 10.6004/jnccn.2011.0077

[18] Kaito K , Kobayashi M, Katayama T, et al Long-term administration of G-CSF for aplastic anaemia is closely related to the early evolution of monosomy 7 MDS in adults. Br J Haematol. 1998;103:297-303. 9827895 10.1046/j.1365-2141.1998.01014.x

[19] Fine JP , Gray RJ. A proportional hazards model for the subdistribution of a competing risk. J Am Stat Assoc. 1999;94:496-509.

[20] Mollica MA , Tesauro G, Gallicchio L, Guida J, Maher ME, Tonorezos E. Survivorship science at the National Institutes of Health 2017-2021. J Cancer Surviv. 2024;18:1443-1452. 37301792 10.1007/s11764-023-01414-0

[21] Rollison DE , Howlader N, Smith MT, et al Epidemiology of myelodysplastic syndromes and chronic myeloproliferative disorders in the United States, 2001-2004, using data from the NAACCR and SEER programs. Blood. 2008;112:45-52. 18443215 10.1182/blood-2008-01-134858

[22] Petrone G , Gaulin C, Derkach A, et al Routine clinical parameters and laboratory testing predict therapy-related myeloid neoplasms after treatment for breast cancer. Haematologica. 2023;108:161-170. 35770528 10.3324/haematol.2021.280437 PMC9827166

[23] Gibson CJ , Lindsley RC, Gondek LP. Clonal hematopoiesis in the setting of hematopoietic cell transplantation. Semin Hematol. 2024;61:9-15. 38429201 10.1053/j.seminhematol.2024.01.011 PMC10978245

[24] Kishtagari A , Corty RW, Visconte V. Clonal hematopoiesis and autoimmunity. Semin Hematol. 2024;61:3-8. 38423847 10.1053/j.seminhematol.2024.01.012

[25] Pagliuca S , Ferraro F. Immune-driven clonal cell selection at the intersection among cancer, infections, autoimmunity and senescence. Semin Hematol. 2024;61:22-34. 38341340 10.1053/j.seminhematol.2024.01.002

[26] Ogbue O , Kewan T, Mehkri O, et al Can emergency hematopoiesis due to sepsis lead to clonal hematopoiesis? Implications for understanding the pathogenesis of clonal evolution. Blood. 2023;142:2691.

[27] Kuzmanovic T , Horvath D, Slaughter M, et al Identification and management of Clonal Hematopoiesis of Indeterminate Potential (CHIP) in cancer survivors: The Cleveland Clinic experience. J Clin Oncol. 2023;41:7010-7010.

[28] Weeks LD , Niroula A, Neuberg D, et al Prediction of risk for myeloid malignancy in clonal hematopoiesis. NEJM Evid. 2023;2:EVIDoa2200310. 10.1056/evidoa2200310 PMC10361696 37483562

[29] Singh A , Mencia-Trinchant N, Griffiths EA, et al Mutant PPM1D- and TP53-driven hematopoiesis populates the hematopoietic compartment in response to peptide receptor radionuclide therapy. J Clin Oncol Precision Oncol. 2022;6:e2100309. 10.1200/PO.21.00309 PMC8769150 35025619

[30] Harris PA , Taylor R, Minor BL, et al; REDCap Consortium. The REDCap Consortium: building an international community of software platform partners. J Biomed Inform. 2019;95:103208. 31078660 10.1016/j.jbi.2019.103208 PMC7254481

[31] Harris PA , Taylor R, Thielke R, Payne J, Gonzalez N, Conde JG. Research electronic data capture (REDCap)—a metadata-driven methodology and workflow process for providing translational research informatics support. J Biomed Inform. 2009;42:377-381. 18929686 10.1016/j.jbi.2008.08.010 PMC2700030

