# Supplementary material for: Therapy-Related-Myeloid-Neoplasm-Risk Score: a convenient score for therapy-related myeloid neoplasms risk assessment in adult cancer patients
Source: JNCI Cancer Spectr. 2025 Sep 16;9(5):pkaf087. doi: 10.1093/jncics/pkaf087 (PMC12574320; doi:10.1093/jncics/pkaf087)
Supplement: pkaf087_Supplementary_Data [file pkaf087_supplementary_data.docx]

Supplementary material

Methods

Patients were categorized into cancer-specific risk groups (low, intermediate, high) based on their cancer-specific risk score in the training cohort, which were tested in the validation cohort. More specifically, breast and bladder cancer patients with a score <1 were grouped as low-risk, a score of 1 to <2 were grouped as intermediate risk and ≥2 had high-risk. Prostate cancer patients with a score <3 were grouped as low-risk, a score of 3 to <5.5 were grouped as intermediate-risk and ≥5.5 had high-risk. GI cancer patients were similar except those patients with a score of ≥4.5 were considered high-risk. Lung cancer patients with a score <1.5 were considered low-risk, a score of 1.5 to <3.5 were grouped as intermediate risk and ≥3.5 had high-risk. Because of the variable frequencies in scores within each cancer, different cut points were used to account for frequencies within each score and the range of scores within each cancer. For example, the incidence of tMN in breast cancer patients with a risk score of 0 was 0.27% and those with a risk score of 0.5 was 0.26%, so they were assigned “low-risk” due to similar incidence of tMN. Whereas breast cancer patients with a risk score of 1.0 and 1.5 had similar incidence of tMN (0.45 and 0.49%, respectively), but this was distinct from the low risk (i.e. almost double the risk) and the high-risk patients (incidence of tMN was ≥0.9% in patients who had risk scores of ≥2). Other cancers were evaluated similarly.

Tables

Table S1**:** Definitions of each cancer cohort by morphology and topography codes

| **Cancer Type** | **Morphology Codes** | **Topography Codes** |
| --- | --- | --- |
| Melanoma | 8720-8790 | C440-C449 |
| Renal cell | all excluding 9050-9055, 9140, 9590-9989 | C649 |
| Head and neck | excluding 9050-9055, 9140, 9590-9992 | C000-009,019-069,079-119, 129-140, 142, 148 |
| Gastrointestinal (GI) | excluding 9050-9055, 9140, 9590-9992 | C150-189, 199, 209-212, 218, 220-221, 239-259, 260, 268-269, 480-482, 488 |
| Lung and Bronchus | excluding 9050-9055, 9140, 9590-9992 | C340-C349 |
| Breast | excluding 9050-9055, 9140, 9590-9992 | C500-C509 |
| Uterine | excluding 9050-9055, 9140, 9590-9992 | C540-C549, C559 |
| Prostate | excluding 9050-9055, 9140, 9590-9992 | C619 |
| Bladder Cancer | excluding 9050-9055, 9140, 9590-9992 | C670-C679 |
| tMDS | 9980, 9982-9983, 9985-9986, 9989, 9991-9992 | all topography codes |
| tAML | 9727, 9840, 9861, 9865-9867, 9869-9874, 9891, 9895-9898, 9910-9911, 9920, 9930-9931, 9984, 9987 | all topography codes |

Table S2**:** Codes for Transplant

| **Time Period** | **ICD-9codes (diagnosis codes found in MEDPAR, NCH, & OUTPT files)** | **ICD-9 procedure codes** | **ICD-10 codes (diagnosis codes found in MEDPAR, NCH, & OUTPT files)** | **ICD-10 procedure codes** | **DRG codes (found in MEDPAR files)** | **Medpar transplant indicator code (transplant)** | **CPT/HCPCS codes (procedure codes found in NCH and OUPT files)** |
| --- | --- | --- | --- | --- | --- | --- | --- |
| **Solid Organ*** Prior to 1/1/2007 | V42.0-V42.l, V42.6-V42.7, V42.83-V42.84, 996.81-996.84, 996.86-996.87 |  |  |  | 302, 480, 495, 512, 513 | 2, 7 | 44136, 47135-47136, 48160, 50360, 50365, 32851-32854, 33935, 33945 |
| **Solid Organ*** 2007 | V42.0-V42.l, V42.6-V42.7,  V 42.83-V 42.84, 996.81-996.84, 996.86-996.87 |  |  |  | - | 2, 7 | 44136, 47135-47136, 48160, 50360, 50365, 32851-32854, 33935, 33945 |
| **Solid Organ*** After 12/31/2007 | V42.0-V42.l, V42.6-V42.7,  V 42.83-V 42.84, 996.81-996.84, 996.86-996.87 |  | Z94 |  | 005-008, 010, 652 | 2, 7 | 44136, 47135-47136, 48160, 50360, 50365, 32851-32854, 33935, 33945 |
| **HCT** | V4281 | 4102, 4103, 4105, 4106, 4108, 4101, 4104, 4107, 4109,4100 | Z9481 | 30230G3, 30230G4, 30230Y4, 30230X0, 30230Y3, 30230G0, 30230AZ, 30230Y0 | 481 | 2 | 38240, S2142, 38241 |

Abbreviations: HCT – hematopoietic cell transplant, NOS – not otherwise specified.

* Since the DRG codes changed in 2007, the specific codes both before and after this time were captured.

**If a patient has a code for any transplant complications (i.e. ICD-9 codes: 99685, 27950-27953), they were included as a 'yes' for transplant.

Table S3: ICD-9 and 10 Codes of Covariates

| **Condition** | **ICD-9 CM code** | **ICD-10 CM code** |
| --- | --- | --- |
| Hypertension | 7962, 4019, 40200, 40201, 40210, 40211, 40290, 40291 | R03.0, I10, I11.9, I11.0 |
| Heart disease | 41000-41002, 41010-41012 41020-41022, 41030-41032, 41040-41042, 41050-41052, 41060-41062, 41070-41072, 41080-41082, 41090-41092, 4110-4111, 41181, 41189, 412, 4130-4131, 4139, 41400-41407, 4142-4144, 4148-4149, 4299 | I21.09, I21.19, I21.11, I21.29, I21.4, I21.3, I24.1, I20.0, I24.0, I24.8, I25.2, I20.8, I20.1, I20.8, I25.10, I25.810, I25.811, I25.812, I25.82, I25.83, I25.84, I25.5, I25.9, I51.9 |
| Stroke | 43300-43301, 43310-43311, 43320-43321, 43330-43331, 43380-43381, 43390-43391, 43400-43401, 43410-43411, 43490-43491, 4350-4353, 4358-4359, 436, 4370, 4378-4379 | I65.1, I63.22, I65.29, I63.139, I65.09, I63.019, I65.8, I63.59, I65.8, I63.59, I65.9, I63.20, I66.09, I63.30, I66.09, I66.9, I63.50, G45.0, G45.8, G45.1, G45.9, I67.89, I67.2, I67.89, I67.9 |
| Atherosclerotic arterial disease | 4400-4401, 44020-44024, 44029-44032, 4404, 4408-4409 | I70.0, I70.1, I70.209, I70.219, I70.229, I70.25, I70.269, I70.299, I70.399, I70.499, I70.599, I70.92, I70.8, I70.90 |
| Diabetes | 25001, 25003, 25011, 25013, 25021, 25023, 25031, 25033, 25041, 25043, 25051, 25053, 25061, 25063, 25071, 25073, 25081, 25083, 25091, 25093, 24900-24901, 24910-24911, 24920-24921, 24930-24931, 24940-24941, 24950-24951, 24960-24961, 24970-24971, 24980-24981, 24990-24991, 25000, 25002, 25010, 25012, 25020, 25022, 25030, 25032, 25040, 25042, 25050, 25052, 25060, 25062, 25070, 25072, 25080, 25082, 25090, 25092 | E109, E1065, E1010, E1069, E1011, E10641, E1029, E1021, E10311, E10319, E1036, E1037X1, E1037X1, E1037X2, E1037X3, E1037X9, E1039, E10311, E10319, E1036, E1039, E1040, E1051, E10618, E10620, E10621, E10622, E10628, E10630, E10638, E10649, E108, E108, E089, E099, E139, E0865, E0965, E0810, E0910, E1310, E0810, E0910, E0865, E0801, E0901, E1300, E0811, E08641, E0911, E09641, E1311, E13641, E0965, E0821, E0921, E08311, E08319, E0836, E0837X1, E0837X2, E0837X3, E0837X9, E0839, E09311, E09319, E0936, E0937X1, E0937X2, E0937X3, E0937X9, E0939, E1339, E0840, E0841, E0842, E0843, E0844, E0849, E08610, E0940, E0941, E0942, E0943, E0944, E0949, E09610, E1340, E1341, E1342, E1343, E1344, E1349, E0851, E0951, E1359, E08618, E08620, E08621, E08622, E08628, E08638, E0869, E09618, E09620, E09621, E09622, E09628, E09630, E09638, E09649, E0969, E13620, E13621, E13622, E13628, E13638, E13649, E1365, E1369, E088, E098, E138, E119, E1165, E1310, E1169, E1100, E1101, E1165, E11641, E1129, E1121, E11311, E11319, E1136, E1139, E1140, E1151, E11618, E11620, E11621, E11622, E11628, E11630, E11638, E11649, E118 |
| Infections (plus those below) | 0419, 042 | B9689, B20 |
| Hepatitis | 07022-07023, 07030-07033, 07041-07044, 07049, 07051-07054, 07059, 0706, 07070-07071 | B181, B180, B169, B1910, B161, B1711, B170, B172, B182, B178, B1710, B188, B189, B190, B1920, B1921 |
| Other viruses | 07951-07953, 07959, 0796, 07981-07983, 0785 | B9733, B9734, B9735, B333, B9739, B974, B334, B9721, B343, B259 |
| Pneumonia | 4800-4803, 4808-4809, 481, 4820-4822, 48230-48232, 48239-48242, 48249, 48281-48284, 48289, 4829-4831, 4838, 4841, 4843, 4845-4848, 485-486, 4870-4871, 4878, 48801-48802, 48809, 48811-48812, 48819, 48881-48882, 48889, 490, 4910-4911, 49120-49122, 4918-4919 | J120, J121, J122, J1281, J1289, J129, J13, J181, J150, J151, J14, J154, J153, J1520, J15211, J15212, J1529, J158, J155, J156, A481, J159, J157, J160, J168, B250, A3791, A221, B440, J17, J180, J189, J1100, J129, J101, J111, J112, J1181, J1189, J09X1, J09X2, J09X3, J09X9, J1008, J101, J40, J410, J411, J449, J441, J440, J418, J42 |
| Septicemia | 0380, 03810-03812, 03819, 0382-0383, 03840-03844, 03849, 0388 | A409, A412, A4101, A4102, A411, A403, A414, A4150, A413, A4151, A4152, A4153, A4159, A4189 |
| Intestinal infections | 00800-00804, 00809, 0081-0083, 00841-00847, 00849, 0085, 00861-00867, 00869, 0088, 0090-0093 | A044, A040, A041, A042, A043, A048, A045, A046, A047, A049, A080, A082, A0811, A0819, A0831, A0832, A0839, A088, A09 |
| Kidney/bladder infection | 59010-59011, 5902-5903, 59080-59081, 5909, 5950-5954, 5959, 5990 | N10, N151, N2884, N12, N16, N159, N3000, N3010, N3020, N3030, N3080, N3090, N390 |
| Tick borne | 0871, 0879-0880, 08881-08882, 08240-08241, 08249, 0828-0829 | A681, A689, A449, A6920, B600, A7740, A7741, A7749, A778, A799 |
| Growth factor use; HCPCS codes** | J1440-2, J1446-7, Q5101, J2505, J2820 | J1440-2, J1446-7, Q5101, J2505, J2820 |
| Acute autoimmune diseases | 136.1, 242.0, 245.2, 281.0, 283.0, 287.31, 364.3, 379.00, 374.53 | M35.2, E05.0, E06.3, D51.0, D59.1, D69.3, H20.9, H15.099, H02.739 |
| Chronic autoimmune diseases | 135, 255.41, 340, 357.0, 357.81, 358.0, 446.0, 446.4, 446.5, 555, 556, 571.42, 571.6, 579.0, 695.4, 696.0-696.1, 701.0, 709.01, 710, 710.0, 710.1, 710.2, 710.3, 710.4, 714.0-714.2, 714.8, 714.3, 720.0, 725 | D86.9, E27.1, G35, G61.0, G61.81, G70.0, M30.0, M31.30, M31.6, K50, K51, K75.4, K74.3, K90.0, L93.0, L40, L94.0, L80, M32.10, M34, M35.0, M33.1, M33.2, M05, M06, M08, M45, M35.3 |

| Table S4: Classification of Autoimmune Conditions Based on Immunological Status and Duration of Immunosuppression | | | |
| --- | --- | --- | --- |
| Condition | Deemed non-autoimmune | Deemed autoimmune with relatively longer duration of immunosuppression | Deemed autoimmune with shorter duration of immunosuppression |
| Autoimmune conditions (ICD-9 CM codes below) | 099.3; 242 (includes all causes of thyrotoxicosis: graves disease, toxic nodular goiter, toxic thyroid nodule, and lymphocytic thyroiditis); 281 (2810 Pernicious anemia already included) 357; 579 | 135.X; 245.2; 255.41; 340.X; 358; 446; 446.4; 446.5; 555.X; 556.X; 571.42; 571.6; 579.0; 695.4; 696.0-696.1; 701.0; 710; 710.1-710.4; 714; 720; 725.X | 136.1; 283; 287.31; 357.1; 364.3; 379; 374.53; 709.01; 725.X; 2810; 245.2 |
| ICD-9 CM code: 099.3 (ICD-10-CM M02.30 Reiter's disease), 135.X (ICD-10-CM D86.9 Sarcoidosis, unspecified), 136.1 (ICD-10-CM M35.2 Behçet's disease), 242 (Thyrotoxicosis with or without goiter), 245.2 (ICD-10-CM E06.3 Autoimmune thyroiditis), 255.41 (ICD-10-CM E27.1 Primary adrenocortical insufficiency), 281 (Other deficiency anemias, 281.0=Pernicious anemia), 283 (Acquired hemolytic anemias;283.0 Autoimmune hemolytic anemias), 287.31 (ICD-10-CM D69.3 Immune thrombocytopenic purpura), 340.X (ICD-10-CM G35 Multiple sclerosis), 357 (Inflammatory and toxic neuropathy: 357.1: Polyneuropathy due to collagen vascular disease) , 358 (Myoneural disorders; MG,Lambert Eaton Syndrome) , 364.3 (ICD-10-CM H20.9 Unspecified iridocyclitis) , 379 (Other disorders of eye including scleritis, episcleritis), 374.53 ( ICD-10-CM H02.739 Hypopigmentation of eyelid), 446 (Polyarteritis nodosa and allied conditions), 446.4 (ICD-10-CM M31.30 Wegener's granulomatosis without renal involvemen), 446.5 (ICD-10-CM M31.6 Other giant cell arteritis), 555.X(Regional enteritis of SI, LI, unspecified sites) -556.X (UC), 571.42 (ICD-10-CM K75.4 Autoimmune hepatitis), 571.6 (Biliary cirrhosis (PBC SBC)), 579 (All causes of Intestinal malabsorptions, 579.0: Celiac disease) , 695.4 (ICD-10-CM L93.0 Discoid lupus erythematosus), 696.0-696.1 (Psoriasis and similar disorders), 701.0 (ICD-10-CM L94.0 Localized scleroderma ), 709.01 (Vitiligo), 710 (Diffuse diseases of connective tissue, 710.0 SLE ), 710.1-710.4 (710.1=Systemic sclerosis, 710.2:Sicca synd, 710.3:Dermatomyositis, 710.4:Polymyositis), 714 ( Rheumatoid arthritis and other inflammatory polyarthropathies), 720 (Ankylosing spondylitis and other inflammatory spondylopathies), 725.X (Polymyalgia rheumatica), 2810 (pernicious anemia- already included under 281) | | | |

| Table S5: Patient characteristics of first primary solid cancers diagnosed 2000-2011 using SEER-Medicare | | | | | | |
| --- | --- | --- | --- | --- | --- | --- |
|  |  | Training cohort | |  | Validation cohort | |
|  |  | n=582,234 | |  | n=388,156 | |
| Characteristics | | n | % |  | n | % |
| **Age at first primary cancer** | |  |  |  |  |  |
|  | <70 years | 156958 | 27% |  | 104308 | 27% |
|  | 70-<75 years | 178264 | 31% |  | 118742 | 31% |
|  | ≥75 years | 247012 | 42% |  | 165106 | 43% |
| **Sex** | |  |  |  |  |  |
|  | Male | 334449 | 57% |  | 222669 | 57% |
|  | Female | 247785 | 43% |  | 165487 | 43% |
| **Race** | |  |  |  |  |  |
|  | White | 496753 | 85% |  | 331132 | 85% |
|  | Black | 49688 | 9% |  | 33163 | 9% |
|  | Other | 31345 | 5% |  | 20943 | 5% |
|  | Unknown | 4448 | 1% |  | 2918 | 1% |
| **Census Tract Poverty Indicator** | |  |  |  |  |  |
|  | 0-<5% | 161780 | 28% |  | 107937 | 28% |
|  | 5-<10% | 162439 | 28% |  | 108249 | 28% |
|  | 10-<20% | 157919 | 27% |  | 105446 | 27% |
|  | 20-100% | 98406 | 17% |  | 65408 | 17% |
|  | Unknown | 1690 | 0% |  | 1116 | 0% |
| **First Primary Cancer** | |  |  |  |  |  |
|  | Lung | 60328 | 10% |  | 40308 | 10% |
|  | Breast | 104057 | 18% |  | 69697 | 18% |
|  | Head and neck | 12272 | 2% |  | 8193 | 2% |
|  | Gastrointestinal | 110647 | 19% |  | 74164 | 19% |
|  | Kidney, renal, and pelvis | 18395 | 3% |  | 11814 | 3% |
|  | Melanoma | 26484 | 5% |  | 17686 | 5% |
|  | Uterine | 19197 | 3% |  | 12820 | 3% |
|  | Ovarian | 7841 | 1% |  | 5220 | 1% |
|  | Prostate | 179487 | 31% |  | 119447 | 31% |
|  | Bladder | 43526 | 7% |  | 28807 | 7% |
| **tMN** | |  |  |  |  |  |
|  | No tMN | 579798 | 99.6% |  | 386492 | 99.6% |
|  | tMN | 2436 | 0.4% |  | 1664 | 0.4% |
| **Chemotherapy** | |  |  |  |  |  |
|  | No chemotherapy | 478190 | 82% |  | 318637 | 82% |
|  | Chemotherapy | 104044 | 18% |  | 69519 | 18% |
| **Radiation** | |  |  |  |  |  |
|  | No radiation | 441224 | 76% |  | 293842 | 76% |
|  | Radiation | 141010 | 24% |  | 94314 | 24% |
| **Stage of first primary cancer diagnosis** | | |  |  |  |  |
|  | Localized/regional | 515036 | 88% |  | 343249 | 88% |
|  | Advanced | 47361 | 8% |  | 31472 | 8% |
|  | Unknown | 19837 | 3% |  | 13435 | 3% |
| **Acute autoimmune disorder** | |  |  |  |  |  |
|  | No acute autoimmune disorder | 548983 | 94% |  | 365914 | 94% |
|  | Acute autoimmune disorder | 33251 | 6% |  | 22242 | 6% |
| **Chronic autoimmune disorder** | |  |  |  |  |  |
|  | No chronic autoimmune disorder | 506884 | 87% |  | 337672 | 87% |
|  | Chronic autoimmune disorder | 75350 | 13% |  | 50484 | 13% |
| **Infection** | |  |  |  |  |  |
|  | No infection | 246616 | 42% |  | 164156 | 42% |
|  | Infection | 335618 | 58% |  | 224000 | 58% |
| **Cardiovascular disease** | |  |  |  |  |  |
|  | No cardiovascular disease | 100171 | 17% |  | 66335 | 17% |
|  | Cardiovascular disease | 482063 | 83% |  | 321821 | 83% |
| **Growth factor** | |  |  |  |  |  |
|  | No growth factor | 567032 | 97% |  | 377967 | 97% |
|  | Growth factor | 15202 | 3% |  | 10189 | 3% |
| **Year of first primary cancer diagnosis** | | |  |  |  |  |
|  | 2000-2003 | 199724 | 34% |  | 133376 | 34% |
|  | 2004-2007 | 190830 | 33% |  | 126606 | 33% |
|  | 2008-2011 | 191680 | 33% |  | 128174 | 33% |
| **Average annual number of physician visits** | | | |  |  |  |
|  | <102 | 132512 | 23% |  | 88335 | 23% |
|  | 102-<587 | 132433 | 23% |  | 88488 | 23% |
|  | 587-<1086 | 150288 | 26% |  | 99959 | 26% |
|  | ≥1086 | 167001 | 29% |  | 111374 | 29% |
| **Duration of Medicare coverage** | |  |  |  |  |  |
|  | 2-<136 months | 105582 | 18% |  | 70217 | 18% |
|  | 136-<187 months | 146777 | 25% |  | 97771 | 25% |
|  | 187-<239 months | 160876 | 28% |  | 107142 | 28% |
|  | ≥239 months | 168999 | 29% |  | 113026 | 29% |
| Abbreviation: tMN - therapy-related myeloid neoplasm. | | | | | | |

| Table S6: Risk of tMN after first primary solid cancers diagnosed 2000-2011 using SEER-Medicare | | | | | | | |
| --- | --- | --- | --- | --- | --- | --- | --- |
|  |  | **tMN** | **No tMN** | **HR*** | **95% CI** | | **p-value** |
|  |  | n=2,426 | n=578,862 |  |  |  |  |
| **Age at first primary cancer** | |  |  |  |  |  | <.0001 |
|  | <70 years | 522 | 156206 | ref |  |  |  |
|  | 70-<75 years | 755 | 177226 | 1.62 | (1.44 | , 1.82) |  |
|  | ≥75 years | 1149 | 245430 | 2.50 | (2.23 | , 2.80) |  |
| **Sex** | |  |  |  |  |  | <.0001 |
|  | Female | 797 | 246988 | ref |  |  |  |
|  | Male | 1629 | 331874 | 1.56 | (1.35 | , 1.79) |  |
| **Race** | |  |  |  |  |  | <.0001 |
|  | Black and unknown | 154 | 53899 | ref |  |  |  |
|  | Other | 2272 | 524963 | 1.49 | (1.26 | , 1.76) |  |
| **Census Tract Poverty Indicator** | | |  |  |  |  | <.0001 |
|  | 0-<5%, unknown | 826 | 162346 | ref |  |  |  |
|  | 5-100% | 1600 | 416516 | 1.26 | (1.16 | , 1.37) |  |
| **First primary cancer** | |  |  |  |  |  | <.0001 |
|  | Lung | 185 | 60143 | ref |  |  |  |
|  | Breast | 406 | 102705 | 2.24 | (1.84 | , 2.74) |  |
|  | Head and neck | 44 | 12228 | 1.23 | (0.89 | , 1.72) |  |
|  | Gastrointestinal | 345 | 110302 | 1.26 | (1.05 | , 1.51) |  |
|  | Kidney, renal, pelvis | 58 | 18337 | 1.65 | (1.22 | , 2.22) |  |
|  | Melanoma | 104 | 26380 | 1.94 | (1.52 | , 2.48) |  |
|  | Uterus | 62 | 19135 | 1.82 | (1.35 | , 2.47) |  |
|  | Ovarian | 33 | 7808 | 2.49 | (1.69 | , 3.66) |  |
|  | Prostate | 998 | 178489 | 2.24 | (1.89 | , 2.67) |  |
|  | Bladder | 191 | 43335 | 1.62 | (1.31 | , 1.99) |  |
| **Chemotherapy or radiation & growth factor** | |  |  |  |  |  | <.0001 |
|  | No chemo/rad (± growth factor) | 1208 | 382860 | ref |  |  |  |
|  | Chemo/rad but no growth factor | 1030 | 181580 | 2.00 | (1.82 | , 2.20) |  |
|  | Chemo/rad/growth factor | 188 | 14422 | 5.98 | (5.04 | , 7.09) |  |
| **Stage of first primary cancer diagnosis** | | |  |  |  |  | <.0001 |
|  | Advanced | 108 | 47201 | ref |  |  |  |
|  | Localized/regional/unknown | 2318 | 531661 | 2.73 | (2.24 | , 3.33) |  |
| **Acute autoimmune disorder** | |  |  |  |  |  | <.0001 |
|  | No autoimmune disorder | 2144 | 545939 | ref |  |  |  |
|  | Autoimmune disorder | 282 | 32923 | 2.05 | (1.83 | , 2.31) |  |
| **Chronic autoimmune disorder** | | |  |  |  |  | <.0001 |
|  | No autoimmune disorder | 1963 | 504070 | ref |  |  |  |
|  | Autoimmune disorder | 463 | 74792 | 1.80 | (1.63 | , 1.98) |  |
| **Cardiovascular disease** | |  |  |  |  |  | 0.0004 |
|  | Cardiovascular disease | 471 | 99529 | ref |  |  |  |
|  | No cardiovascular disease | 1955 | 479333 | 1.21 | (1.09 | , 1.35) |  |
| **Infection** | |  |  |  |  |  | 0.0001 |
|  | No infection | 935 | 245224 | ref |  |  |  |
|  | Infection | 1491 | 333638 | 1.23 | (1.11 | , 1.36) |  |
| Abbreviation: tMN - therapy-related myeloid neoplasm. | | | | | | | |
| *HRs were additionally controlled for year of first primary cancer diagnosis, average annual number of physician visits, and duration of Medicare coverage. | | | | | | | |
